# Satisfaction with Surgical Procedures and Bladder Management of Chronic Spinal Cord Injured Patients with Voiding Dysfunction Who Desire Spontaneous Voiding

**DOI:** 10.3390/jpm12101751

**Published:** 2022-10-21

**Authors:** Shu-Yu Wu, Hann-Chorng Kuo

**Affiliations:** 1 Department of Urology, Taipei Tzu Chi Hospital, Buddhist Tzu Chi Medical Foundation, New Taipei City 231016, Taiwan; 2 Department of Urology, School of Medicine, Tzu Chi University, Hualien 970374, Taiwan; 3 Department of Urology, Hualien Tzu Chi Hospital, Buddhist Tzu Chi Medical Foundation, Hualien 970473, Taiwan

**Keywords:** spinal cord injury, voiding dysfunction, bladder management, patient satisfaction, bladder outlet operation

## Abstract

We aimed to investigate treatment outcome and satisfaction with bladder outlet surgeries and bladder management in patients with spinal cord injury (SCI), voiding dysfunction, and to seek a spontaneous voiding or reflex voiding program. A total of 261 patients were included in this retrospective study. The mean age at surgical procedure was 49.2 ± 15.9 years; the median follow-up period was 11 (IQR 6, 17) years; 119 received a urethral Botox injection, 41 underwent transurethral incision of the bladder neck (TUI-BN), 77 underwent transurethral incision or resection of the prostate (TUI-P or TUR-P), and 24 had an external sphincterotomy. Satisfactory surgical outcome was reported by 80.5% of patients undergoing TUI-BN, 70.8% undergoing external sphincterotomy, 64.9% receiving TUI-P or TUR-P, and 59.7% receiving the urethral Botox injection. Persistent dysuria was the most common reason for dissatisfaction after the urethral Botox injection (73.1%) and TUI-BN (58.5%). Recurrent urinary tract infection continued in most patients after any type of surgery (all >75%). Most patients with SCI were satisfied with their initial bladder outlet operation in facilitating spontaneous voiding. However, repeat, or multiple surgical interventions were needed in 65.5% of SCI patients to achieve satisfactory voiding. A correct diagnosis is very important before every intervention and bladder management to reach the best satisfaction. VUDS is suggested before surgical procedures to ensure efficacy, even in patients with the same level of SCI.

## 1. Introduction

Voiding dysfunction is a common problem in patients with chronic spinal cord injury (SCI) due to neurogenic lower urinary tract dysfunction (NLUTD), which refers to abnormal or difficult function of the bladder and urethra, in individuals with clinically conformed relevant neurologic disorders [1,2]. Patients with different levels of SCI may have different storage and voiding problems. According to urodynamic observations, the NLUTD may occur during bladder filling, including bladder sensation, bladder capacity and detrusor function; or during the voiding phase, including detrusor function and sphincter function [1,2,3]. Different voiding dysfunction has been noted in patients with different SCI levels. However, not all patients with the same level of SCI have the same symptoms. The management of NLUTD in patients with chronic SCI is difficult due to the complexity of, and changes in, different patient conditions [4].

The primary goal of NLUTD management should be the preservation of renal function, which requires prevention of urinary tract infections (UTIs) as well as adequate emptying of the bladder [5]. To achieve these goals, patients with SCI are always managed with indwelling catheterization in the acute stage. About one third of SCI patients continue to use indwelling urethral catheterization as the long-term strategy for bladder management [5]. Intermittent catheterization (IC) has been recommended as the preferred form of bladder management, which allows the achievement of an adequate low-pressure bladder and acceptable bladder capacity [6,7,8]. Some SCI patients with frequent detrusor overactivity (DO) need antimuscarinics to achieve a low-pressure bladder. These overactive bladder inhibitory medications might have side effects that SCI patients could not tolerate, such as constipation, dry mouth, or blurred vision [6]. The most common reasons to stop IC are still related to patient preference and physician recommendation. Patients may dislike IC due to inconvenience in working or tetraplegia and the lack of a care giver. In the case of frequent UTIs, hydronephrosis, and severe urinary incontinence, the physician will advise the patient to change to an indwelling catheter if IC is not suitable for SCI patients [9]. However, the rate of this conversion from IC to indwelling urethral catheter is limited to a small percentage of SCI patients.

SCI patients usually demand spontaneous urination either by bladder reflex triggering or bladder expression. These bladder managements might endanger renal function if the intravesical pressure is not carefully monitored and patients do not follow the standard operation procedure by the physician. Therefore, a surgical procedure to reduce bladder outlet resistance as well as intravesical pressure is indicated. In addition, a condom catheter or pads may be needed in those with complete SCI. Several operations have been recommended to facilitate reflex voiding in patients with chronic SCI. The aim of the procedure is in reducing bladder outlet resistance. The common reported target organs include urethral sphincter, bladder neck, and the prostate [10]. The injection of botulinum toxin A (Botox) into the urethral sphincter can provide muscle relaxation and facilitate reflex voiding [11]. In current evidence, external sphincterotomy can improve dysreflexia symptoms, residual urine volume, and reduce the rate of UTIs, especially in high cervical SCI patients who cannot perform CIC [12]. An opposite report was also presented that the high rate of recurrent symptomatic UTIs, recurrent DSD, or upper tract dilatation, eventually ensued in 68% of patients [13]. In a more recent report, maximum bladder pressure is maintained at a low level over 20 years. However, NDO gradually decreases over time [14]. A bladder neck and prostate operation can also lower bladder outlet resistance and preserve the external sphincter function, which avoids total incontinence [15]. However, real-life reports on long-term patient satisfaction and subsequent bladder management are still lacking. The aim of this study is to report the first large series of SCI patients, which focuses on satisfaction with surgical procedures and bladder management to treat their voiding dysfunction.

## 2. Materials and Methods

### 2.1. Patients

We retrospectively reviewed the medical and surgical records of all SCI patients (including both male and female patients) with voiding dysfunction who received bladder outlet surgery and were followed up at a tertiary teaching hospital during the period of 1997–2020. All patients presented with voiding dysfunction of different severity (from severe dysuria to complete urinary retention) before surgery, and all patients underwent the surgical procedure because their voiding problems (Table 1) were refractory to conservative therapy (such as oral medications, including alpha-blockers) and IC, or they wished to try a catheter to achieve a better life quality. Therefore, after confirming the patient’s wishes and safety, individualized bladder outlet operations were given according to their urodynamic observations.

All patients underwent a physical examination, and their histories, especially the causes of NLUTD and voiding problems, were recorded in detail. A video-urodynamic study (VUDS) was performed before every surgical procedure for all patients. The correct diagnosis, to know the bladder and bladder outlet conditions, bladder compliance, and the presence or absence of vesicoureteral reflux, is important before every treatment for SCI patients, especially before an invasive operation. VUDS provide the best way to the patients’ lower urinary tract condition, and was routinely performed before Botox injection and invasive surgery. The surgical procedure was selected after a comprehensive evaluation of each patient’s subjective symptoms and objective urodynamic findings. We did not record the detail of the post-operative urodynamic study; however, most patients were regularly followed up and the renal condition (hydronephrosis, recurrent pyelonephritis) and renal function (serum creatinine level, glomerular filtration rate, or renal scan) were also monitored in high-risk patients. The patients’ bladder and voiding condition were thoroughly discussed with them, and informed consent was obtained before the operation. All patients were well educated and patients understood that concomitant with the surgical treatment plan, bladder and urinary management would be required post-operatively. We provided close monitoring and follow-up after the surgery.

### 2.2. Surgical Procedures

According to SCI patients’ bladder and bladder outlet conditions and urodynamic results, they received one of the following procedures: urethral Botox injection; transurethral incision of the bladder neck (TUI-BN); transurethral incision or resection of the prostate (TUI-P or TUR-P); or external sphincterotomy. The indications for each surgical procedure are listed in Table 2.

The urethral Botox injection was administered in the operating room under intravenous general anesthesia. One hundred units of Botox was injected into the external urethral sphincter as reported in previous studies [16,17]. One vial of 100 U Botox was reconstituted with 4 mL of normal saline, thereby achieving a concentration equivalent to 25 U per mL. In total, 1 mL of Botox solution was injected trans-urethrally into the urethral sphincter at 3-, 6-, 9-, and 12-o’clock positions in male patients and trans-cutaneously into the urethral sphincter along the urethral lumen at 1-, 4-, 7-, and 10-o’clock positions around the urethral meatus in female patients.

TUI-BN was performed with a resectoscope and diathermy electrode using a 110-W cutting current, similar to that in our previous reports [18,19]. Double incisions were made at the 5- and 7- o’clock positions at the bladder neck. The bladder smooth muscles were cut deeply until the serosa level was reached to make the bladder neck sufficiently open. After the procedure, a urethral Foley catheter was routinely placed for 48 h with continuous irrigation of the bladder with normal saline.

TUI-P and TUR-P were used only in male patients. The total prostate volume was used to determine the methods of operation. Patients with a total prostate volume < 30 mL usually received TUI-P whereas patients with a total prostate volume ≥ 30 mL received TUR-P. The operations were performed in the same way as we reported in our previous study to create a wide-open prostatic urethra [20]. In the TUI-P procedure, the prostate urethra was incised at 5- and 7- o’clock positions bilaterally from the bladder neck to the level of the verumontanum. After the procedure, a urethral Foley catheter was indwelled for 48 h with continuous normal saline bladder irrigation to avoid blood clots formation and catheter obstruction.

External sphincterotomy was performed with a resectoscope and diathermy electrode using a 110-W cutting current in the same way as other transurethral incision operations [21]. A single incision was made at the 12-o’clock position from the bladder neck, prostatic urethra throughout the membranous urethra and the incision was deepened until the entire external sphincter muscle was incised to the serosa level. After the procedure, a urethral Foley catheter was indwelled with tension and placed for 48 h with continuous normal saline irrigation of the bladder.

### 2.3. Follow-Up

The SCI patients received regular post-operative follow-up in our urology clinic. The patients were requested to report their satisfaction with bladder management or surgery. Objective measures included voiding diary, flow rate and post-void residual urine (PVR), and renal and bladder sonography. PVR and voiding efficiency (including patient reports and an ultrasound of the bladder) were used to evaluate the treatment outcome and the need for secondary intervention. Urodynamic study was not performed routinely, but was recommended if patients had poor outcome or were planning for secondary intervention. Postoperative complications and reasons for dissatisfaction were also assessed. A self-reported 4-point Likert scale (from 0 to 3) was used to measure patients’ global response to surgical outcome: (0) no change, (1) mildly satisfied, (2) moderately satisfied, and (3) very satisfied. A score of 2 or 3 was regarded as indicating that patients were satisfied with the procedure. The scores were compared between different SCI subgroups using chi-square test and Fisher’s exact test. A *p*-value of <0.05 was considered statistically significant. All analyses were performed with the SPSS (IBM Corp. Released 2015. IBM SPSS Statistics for Windows, Version 23.0. Armonk, NY, USA: IBM Corp.).

### 2.4. Research Ethics

This study was approved by the Research Ethics Committee of Hualien Tzu Chi hospital, Buddhist Tzu Chi medical foundation. The clinical trial registration number is IRB 110-033-B.

## 3. Results

### 3.1. Patient Characteristics

A total of 261 consecutive patients (222 men and 39 women) with chronic SCI were included in this study. Among these patients, 30 (11.5%) had already died by the most recent follow-up. Therefore, for each of these deceased patients, we used the data collected at the patient’s most recent follow-up visit before death. The mean age at operation was 49.2 ± 15.9 years and the median follow-up period was 11 (IQR = 6, 17) years, and the longest follow-up period was over 25 years.

### 3.2. Satisfaction with Bladder Management or Urological Surgery

Of the 261 patients, 119 received urethral Botox injection, 41 received TUI-BN, 77 received TUI-P or TUR-P, and 24 received external sphincterotomy. Satisfaction with the surgical procedure of patients with different levels of SCI is shown in Table 3. Most of the patients were highly satisfied with the surgical procedure, with TUI-BN ranked first (80.5%) and external sphincterotomy ranked second (70.8%). The adoption of different surgical procedures is highly dependent on the level of SCI and patients’ voiding dysfunctions and VUDS findings. No statistically significant difference in post-operative satisfaction was found between different operations or SCI levels. External sphincterotomy showed good outcomes in patients with thoracic and lumbar spine injury. Patients receiving urethral Botox injection did not show differences between SCI levels.

The typical VUDS results of SCI patients included NDO, DSD, DA, DU, and intrinsic sphincter deficiency (ISD). Autonomic dysreflexia (AD) occurring during examination was also noted in patients with high-level SCI. There was no significant difference in the rate of satisfaction in surgical procedures between SCI patients with different urodynamic findings. In general, patients with NDO and VUDS-proven bladder outlet dysfunction (bladder outlet obstruction [BND] or DSD) showed high satisfaction in TUI-BN, TUI-P, or TUR-P, whereas patients with DA or DU and a tight external sphincter showed less satisfaction with TUI-P or TUR-P. Patients with DSD showed higher satisfaction (63.8–72%) to urethral Botox injection than patients with DU or DA and a tight external sphincter (43.3%). External sphincterotomy was a relatively rare procedure with which patients with NDO and DSD showed high satisfaction.

### 3.3. Overall Satisfaction with Final Bladder Condition

At the follow-up visit, patients who received a urethral Botox injection (58.8%), TUI-BN (87.8%), TUI-P or TUR-P (68.8%), reported high satisfaction with their bladder and voiding condition, but not those who received external sphincterotomy (45.8%). Table 4 shows rates of satisfaction with the surgical procedures. Although some patients were not satisfied with their bladder and voiding conditions after surgery, they could accept the outcome and did not desire a change in their bladder management. Only a small percentage of patients reported not being satisfied with the surgical procedure (*n* = 13, 5%).

### 3.4. Causes of Dissatisfaction with Bladder Management or Surgical Procedures and Long-Term Complications

The causes of dissatisfaction with the surgical procedures were ascertained for every patient using an open question with multiple-choice answers. Table 5 presents the events reported by the patients. Persistent difficult urination was the most common reason for dissatisfaction reported by patients after urethral Botox injection, TUI-BN, and TUI-P or TUR-P; this was particularly the case for those who received a urethral Botox injection. Increased episodes of urgency urinary incontinence (UUI), or stress urinary incontinence (SUI), and persistent AD, were also reported by a small number of patients after surgery to facilitate spontaneous voiding. Exacerbated UUI or SUI was more commonly reported by patients who received TUI-P or TUR-P. Recurrent UTIs was reported frequently by patients in all groups after surgical procedure.

### 3.5. Additional Treatments and Changes of Bladder Management after Initial Treatment for Voiding Dysfunction

After the initial surgical treatment, SCI patients were regularly followed up at our clinic. Because of persistent difficulties in spontaneous urination or exacerbated UUI or SUI, some patients received other surgical therapy or bladder management interventions for improving the quality of life. Detailed data are shown in Table 6. Other medical treatments were necessary for half the patients who received a urethral Botox injection and external sphincterotomy. A detrusor Botox injection was also needed by each patient group at a similar rate. Bladder augmentation or urinary diversion was performed in 10 patients due to AD or low compliant and contracted bladder. Additional repeat urethral Botox (*n* = 70, 26.8%), TUI-BN (*n* = 25, 9.6%), TUI-P and TUR-P (*n* = 24, 9.2%), and external sphincterotomy (*n* = 14, 5.4%), were necessary during the follow-up period after the initial surgical treatment. A small percentage of patients received a sub-urethral sling to achieve urinary continence (*n* = 5, 1.9%) or converted to cystostomy (*n* = 11, 4.2%), indwelling urethral catheter (*n* = 10, 3.8%), or IC (*n* = 16, 6.1%). Most patients could achieve the therapeutic goal of reflex voiding; the catheter dependence rate was only 14.2%.

## 4. Discussion

This study reports a large series focusing on patient satisfaction in surgical procedures for chronic SCI patients with voiding dysfunction who desired spontaneous voiding. Generally, satisfaction was high (65.5%) in the initial surgical procedures. TUI-BN, TUI-P, TUR-P, and external sphincterotomy, provided higher satisfaction. However, repeat surgery or switching to another surgical procedure was necessary for some patients to facilitate spontaneous voiding. VUDS should always be performed before surgery to ensure efficacy even if different patients have the same level of SCI.

Due to the nature of SCI and NLUTD, patients may experience different LUTD, including urinary incontinence and difficulty in emptying the bladder. Proper bladder management is important to avoid severe complications, such as recurrent UTIs, vesicoureteral reflux, hydronephrosis, urolithiasis, and renal failure [22]. Conservative management for bladder emptying, including IC and indwelling catheter, is often recommended by physicians. However, UTIs and urethral complications are common disadvantages reported during IC [23]. In previous report by Liu et al. [24], only 21% of SCI patients report normal voiding without any other form of bladder management. In the health-related quality of life report, clean IC by attendant, indwelling transurethral catheterization, and indwelling suprapubic catheterization, are the three groups with the worst mental status. Frequent urinary incontinence is reported as the major reason for dissatisfied. Reflex voiding, arising from both physical and psychological considerations, remains the wish of SCI patients. Patients are often willing to try surgical interventions to facilitate reflex voiding for a better quality of life [25,26].

In this study, we report the results of common bladder outlet procedures to facilitate spontaneous voiding, including a urethral Botox injection, TUI-BN, TUIP or TURP, and external sphincterotomy. The core value of all surgical interventions is the reduction of bladder outlet resistance. Among these procedures, the urethral Botox injection is quite different from others in that it mediates the alteration of bladder outlet function rather than anatomy. DSD is used to descript bladder outlet obstruction presenting from involuntary urethral sphincter contraction during detrusor contraction. DSD is related to many neurological diseases including SCI and multiple sclerosis, resulting in urine retention, recurrent UTIs, or upper urinary tract impairments [27]. The diagnosis of DSD often relied on urodynamic study with electromyography, which could be performed with a needle or surface electrodes [28]. The combination of VUDS during the voiding phase can improve the detection of DSD [29]. Current evidence revealed that oral medications are not effective in the treatment of DSD [27]. The urethral Botox injection is widely applied to patients with SCI and DSD who do not desire surgery, or who are not willing to perform IC [30]. Safety and efficacy have been well studied in patients with external urethral sphincter dysfunction [11,31,32]. After urethral Botox injections, patients can experience decreased urethral pressure, decreased post-void residual volume, and reduced AD episodes. A successful outcome was noted in about 60% of cases in previous reports, similar to this study (59.4%) [21,33]. The therapeutic effect of a urethral Botox injection, which focuses on functional change, is also reflected in lower patient satisfaction and the higher rate of subsequent treatment. This may be because the effect is not immediate and tends to wear off over time; therefore, regular repeat injections are mandatory to achieve a persistent effect. While 61.6% of patients who received a urethral Botox injection were satisfied with the outcome, only 25.2% wished to change the procedure for a better treatment result. Besides transurethral injection, Botox could also be applied transperineally with similar effectiveness. In a previous comparative study, no significant difference was noted between the groups in all of the outcome measures [34].

TUI-BN had a very high satisfaction rate among patients with cervical SCI, NDO, AD, or DSD, because they usually had a tight bladder neck and good detrusor contractility [18]. Patients with low-level SCI and DU or DA may also benefit from TUI-BN because they can use abdominal straining to urinate after the bladder neck is incised open [19]. Satisfaction with TUI-P or TUR-P was fair among patients of all SCI levels and patients with NDO, DU, or DA. A similar treatment outcome in our study might have resulted from relief of prostatic urethral resistance; therefore, most patients could urinate spontaneously either by detrusor contractility or abdominal pressure. Patients have NDO at a higher level of SCI (infrapontine-suprasacral) lesions, and develop DU or DA at a lower level of SCI (sacral/infrasacral) lesions [35]. External sphincterotomy was only performed for patients with NDO and DSD. In a more recent study reported by Takahashi et al. [14], 27 out of 37 patients required reflex voiding to a condom catheter, while others need to change their bladder management to suprapubic catheter or IC. The most common cause for the change of bladder management is persistent AD, which was defined as failure by the authors. Lower preoperative bladder pressure was thought to be a poor prognostic factor for external sphincterotomy. Urinary incontinence is the most common cause of treatment dissatisfaction after external sphincterotomy; however, most patients could accept that treatment outcome because all patients had been informed of this possible result before surgical intervention. Perkash had reported the use of external sphincterotomy with laser (including bladder neck and prostate ablation), which could be beneficial to AD control. However, recurrent episodes of AD with difficulty in voiding may need reevaluation in patients with failed external sphincterotomy. Adequate bladder drainage could provide durable relief from AD [12,36]. Although repeat procedures were necessary for some patients, the treatment outcome was always satisfactory. Different bladder conditions play a critical role in the ability to reflex voiding after bladder outlet surgery. For patients with DU or DA, adequate abdominal pressure is necessary to achieve reflex voiding [37].

The commonly reported urological complications in SCI patients include symptomatic UTIs, hydronephrosis, and severe urinary incontinence [5,38]. The problems are mostly related to unresolved NLUTD caused by the SCI. Incomplete drainage with large PVR and high intravesical pressure are risk factors related to these events [39]. To reduce complications and improve quality of life, many efforts are made to facilitate bladder emptying. In addition to IC and medical treatment, bladder outlet operations to reduce bladder outlet resistance are widely used [40]. However, high rates of long-term complications are still noted after the procedures. In this study, the most common complications are recurrent UTIs, difficult urination, and urine incontinence, which are related to the characteristics of SCI itself. Catheter-associated UTIs in long-term care setting is an important issue in the care of SCI patients. The best way to prevent catheter-associated UTIs is to remove the catheter, which is nearly impossible in such patients with chronic SCI. Therefore, non-antibiotic prophylaxis and other preventive methods had been reported, but there is still a lack of strong evidence [41]. In the long-term follow-up, two patients had persistent hydronephrosis even after a urethral Botox injection, indicating that treatment targeting bladder outlet obstruction might not be adequate to reduce high intravesical pressure. In addition, some patients had exacerbated urinary incontinence after the initial surgical procedure, which necessitated further bladder management or another surgical intervention. The reasons for dissatisfaction with surgery tend to be similar. Patients wish and expect to regain reflex voiding without de novo or exacerbated urinary incontinence; however, this expectation might sometimes be unrealistic.

This study has several limitations. First, it was a retrospective study; we used self-reported measures to determine global assessment of satisfaction with outcomes, and some data were incomplete due to time or patient factors. Post-operative PVR is usually an important objective measure. However, unfortunately, patients were usually unable or unwilling to take the ultrasound exam during clinic follow-up. The most common reason is inconvenient to perform IC outside. Due to the disadvantage, we were usually unable to record PVR in the correct timing. Second, some operations were performed according to the patient preference to facilitate reflex voiding, which have affected the overall satisfaction with treatment. Third, the number of patients undergoing different surgical procedures and at different SCI levels varied, which might also have affected the interpretation of results. Fourth, the outcome evaluations were focused on patient report satisfaction and the lack of detailed post-operative urodynamic data, which might cause unpredictable, higher intravesical pressure and might affect the renal function. In fact, the satisfaction of chronic SCI patients after bladder management or urological surgeries varied widely. Patients might desire spontaneous voiding by reflex or Valsalva maneuver, but would complain of urinary incontinence (urgency UI or stress incontinence) after the procedure. Therefore, the aim of this study was to assess the real-life satisfaction of SCI patients to the management of their voiding dysfunction. The satisfaction includes voiding condition and urinary continence. The results of this study could provide evidence for urologists to discuss with SCI patients who are planning to undergo urological procedures to facilitate spontaneous voiding.

## 5. Conclusions

Bladder outlet operations are common procedures to reduce bladder outlet resistance and facilitate reflex voiding. In this study, most patients with chronic SCI and voiding dysfunction were satisfied with the initial surgical procedure. Patients who were not satisfied with the initial surgical procedure needed repeat interventions or switched to another surgical procedure to obtain a satisfactory outcome. Nevertheless, the need of a catheter for bladder emptying can be reduced after surgical intervention, and only a small number of SCI patients required a catheterization for voiding. VUDS is suggested before surgical procedures to ensure efficacy, even when different patients are at the same level of SCI.

## Figures and Tables

**Table 1:** Pre-operative clinical data according to different spinal cord injury levels.

	Total (*n* = 261)	SCI C-Spine (*n* = 137)	SCI T-Spine (*n* = 70)	SCI L-Spine (*n* = 47)	SCI S-Spine (*n* = 7)	*p*-value
**Age at SCI (years)**	42.0 ± 17.5	44.8 ± 17.4	35.6 ± 16.5	43.2 ± 17.8	46.3 ± 13.2	0.005
**Age at operation (year)**	49. 2 ± 15.9	49.7 ± 15.9	45.6 ± 16.4	52.2 ± 13.5	55.9 ± 19.7	0.083
**Follow-up period (years)**	12.0 ± 6.4	12.4 ± 6.4	11.6 ± 6.3	11.7 ± 6.7	11.0 ± 6.3	0.809
***Clinical symptoms***						
**Dysuria (N, %)**	225, 86.2%	119, 86.9%	59, 84.3%	41, 87.2%	6, 85.7%	0.958
**Urine retention (N, %)**	15, 5.7%	3, 2.2%	8, 11.4%	3, 6.4%	1, 14.3%	0.021
**Recurrent UTIs (N, %)**	213, 81.6%	109, 79.6%	57, 81.4%	41, 87.2%	6, 85.7%	0.693
**Hydronephrosis (N, %)**	22, 8.4%	9, 6.6%	10, 14.3%	3, 6.4%	0	0.277
**Difficult IC (N, %)**	1, 0.4%	0	0	1, 2.1%	0	0.207
**Severe AD (N, %)**	74, 28.4%	73, 53.3%	1, 1.4%	0	0	0.000
**Urolithiasis (N, %)**	2, 0.8%	0	0	2, 4.3%	0	0.085
**CKD (N, %)**	1, 0.4%	1, 0.7%	0	0	0	1.000
***VUDS observations***						
**FSF**	153.8 ± 108.3	141.0 ± 96.0	156.7 ± 118.7	174.2 ± 110.0	173.4 ± 79.6	0.008
**FS**	207.7 ± 131.8	192.0 ±117.7	206.9 ± 146.1	243.1 ± 126.7	240.4 ± 78.3	0.000
**US**	232.2 ± 148.1	215.9 ± 135.9	228.0 ± 159.0	276.6 ± 147.0	274.1 ± 96.7	0.000
**Pdet**	29.7 ± 25.1	32.3 ± 22.9	32.3 ± 26.7	18.6 ± 22.2	15.3 ± 24.1	0.000
**Qmax**	4.6 ± 5.7	4.0 ± 5.0	5.1 ± 6.2	4.4 ± 5.6	4.8 ± 6.8	0.101
**Volume**	74.3 ± 108.9	66.4 ± 100.3	75.9 ± 109.4	83.0 ± 119.1	106.6 ± 142.6	0.122
**PVR**	219.8 ± 19.06	224.2 ± 184.0	204.6 ± 187.4	245.2 ± 211.1	244.4 ± 199.1	0.116
**CBC**	293.3 ± 174.3	290.6 ± 167.8	279.7 ± 177.3	325.5 ± 181.9	350.9 ± 152.5	0.011
**VE**	0.31 ± 0.35	0.29 ± 0.38	0.32 ± 0.35	0.31 ± 0.35	0.33 ± 0.43	0.686

SCI, spinal cord injury; C-spine, cervical spine; T-spine, thoracic spine; L-spine, lumbar spine; S-spine, sacral spine; UTIs, urinary tract infections; IC, intermittent catheterization; AD, autonomic dysreflexia; VUDS, video-urodynamic studies; FSF, first sensation of filling; FS, full sensation; US, urge sensation; Pdet, maximum detrusor pressure; Qmax, maximum flow rate; PVR, post-voiding residual urine volume; CBC, cystometric bladder capacity; VE, voiding efficiency.

**Table 2:** Interventional management and surgical procedures for neurogenic lower urinary tract dysfunction in chronic spinal cord injured patients to facilitate spontaneous voiding.

Aims of Treatment	Interventional Procedure	Specific Indications
**To facilitate voiding**	Urethral Botox injection	DSD, AD
	TUI-BN	DU, AD, BND
	TUIP/TURP	DU, NDO with BND, AD
	External sphincterotomy	Quadriplegia, DSD, AD
	Alpha-blocker, baclofen	DSD, AD

AD, autonomic dysreflexia; BND, bladder neck dysfunction; NDO, neurogenic detrusor overactivity; DSD, detrusor sphincter dyssynergia; DU, detrusor underactivity; TUI-BN, transurethral incision of the bladder neck; TUI-P, transurethral incision of the prostate; TUR-P, transurethral resection of the prostate.

**Table 3:** Chronic spinal cord injured patients’ satisfaction with bladder management or urological surgeries for spontaneous voiding. The patients had different levels of injury.

	Satisfied	Total SCI (*n* = 261)	Cervical SCI (*n* = 137)	Thoracic SCI (*n* = 70)	Lumbar and Sacral SCI (*n* = 54)	*p*-value
**Urethral Botox injection (*n* = 119)**	**No** **Yes**	48 71 (59.7%)	17 34 (66.7%)	20 24 (54.5%)	11 13 (54.2%)	0.403
**TUI-BN** **(*n* = 41)**	**No** **Yes**	8 33 (80.5%)	2 19 (90.5%)	5 7 (58.3%)	1 7 (87.5%)	0.098
**TUIP/TURP (*n* = 77)**	**No** **Yes**	27 50 (64.9%)	14 29 (67.4%)	4 9 (69.2%)	9 12 (57.1%)	0.676
**External sphincterotomy** **(*n* = 24)**	**No** **Yes**	7 17 (70.8%)	7 15 (68.2%)	0 1 (100%)	0 1 (100%)	1.000

SCI, spinal cord injury; TUI-BN, transurethral incision of the bladder neck; TUI-P, transurethral incision of the prostate; TUR-P, transurethral resection of the prostate.

**Table 4:** Overall satisfaction with final bladder management and urological surgeries for spontaneous voiding.

	Total SCI (*n* = 261)	Satisfied (*n* = 170)	Acceptable + Unsatisfied (*n* = 91)	*p*-value
**Urethral Botox injection**	119	70 (58.8%)	49 (41.2%)	0.050
**TUI-BN**	41	36 (87.8%)	5 (12.2%)	0.001
**TUIP/TURP**	77	53 (68.8%)	24 (31.2%)	0.417
**External sphincterotomy**	24	11 (45.8%)	13 (54.2%)	0.037

SCI, spinal cord injury; TUI-BN, transurethral incision of the bladder neck; TUI-P, transurethral incision of the prostate; TUR-P, transurethral resection of the prostate.

**Table 5:** Causes of dissatisfaction with bladder management after initial treatment for spontaneous voiding.

	Total (*n* = 261)	Recurrent UTIs (*n* = 214)	Difficult Urination (*n* = 154)	UUI/SUI (*n* = 47)	AD (*n* = 38)
**Urethral Botox injection**	119	99 (83.2%)	87 (73.1%)	15 (12.6%)	17 (14.3%)
**TUI-BN**	41	31 (75.6%)	21 (58.5%)	10 (24.4%)	4 (9.8%)
**TUIP/TURP**	77	65 (84.4%)	41 (53.2%)	19 (24.7%)	12 (15.6%)
**External sphincterotomy**	24	19 (79.2%)	4 (16.7%)	3 (12.5%)	5 (20.8%)

AD, autonomic dysreflexia; SUI, stress urinary incontinence; TUI-BN, transurethral incision of the bladder neck; TUI-P, transurethral incision of the prostate; TUR-P, transurethral resection of the prostate; UTIs, urinary tract infection; UUI, urgency urinary incontinence.

**Table 6:** Changes of bladder management or surgical procedures after initial treatment for voiding dysfunction in chronic spinal cord injury patients.

	Urethral Botox (*n* = 119)	TUI-BN (*n* = 41)	TUIP/TURP (*n* = 77)	External Sphincterotomy (*n* = 24)
Medical treatment	56 (47.1%)	0	0	12 (50%)
Detrusor Botox injection	20 (16.8%)	7 (17.1%)	9 (11.7%)	4 (16.7%)
Urethral Botox injection	42 (35.3%)	9 (22.0%)	16 (20.8%)	3 (12.5%)
TUI-BN	16 (13.4%)	5 (12.2%)	3 (3.9%)	1 (4.2%)
TUIP/TURP	9 (7.6%)	4 (9.8%)	11 (14.3%)	0
External sphincterotomy	4 (3.4%)	1 (2.4%)	1 (1.3%)	8 (33.3%)
Sub-urethral sling	2 (1.7%)	2 (4.9%)	1 (1.3%)	0
IC/CIC	9 (7.6%)	4 (9.8%)	2 (2.6%)	1 (4.2%)
Cystostomy	2 (1.7%)	1 (2.4%)	4 (5.2%)	4 (16.7%)
Indwelling urethral catheter	4 (3.4%)	1 (2.4)	3 (3.9%)	2 (8.3%)

IC, intermittent catheterization; CIC, clean intermittent catheterization; TUI-BN, transurethral incision of the bladder neck; TUI-P, transurethral incision of the prostate; TUR-P, transurethral resection of the prostate.

## Data Availability

Data available on request due to restrictions.
